# Clustering and Switching in Verbal Fluency Across Varying Degrees of Cognitive Control Demands: Evidence From Healthy Bilinguals and Bilingual Patients With Aphasia

**DOI:** 10.1162/nol_a_00053

**Published:** 2021-12-23

**Authors:** Erin Carpenter, Claudia Peñaloza, Leela Rao, Swathi Kiran

**Affiliations:** Aphasia Research Laboratory, Department of Speech, Language and Hearing Sciences, Sargent College of Health & Rehabilitation Sciences, Boston University, Boston, MA, USA

**Keywords:** bilingual aphasia, semantic executive control, language control, lexical access, semantic fluency, phonemic fluency

## Abstract

Different linguistic contexts place varying amounts of cognitive control on lexical retrieval in bilingual speakers, an issue that is complicated in bilingual patients with aphasia (BPWA) due to subsequent language and cognitive deficits. Verbal fluency tasks may offer insight into the interaction between executive and language control in healthy bilinguals and BPWA, by examining conditions with varying cognitive control demands. The present study examined switching and clustering in verbal fluency tasks in BPWA and healthy bilinguals across single- and dual-language conditions. We also examined the influence of language processing and language proficiency on switching and clustering performance across the dual-language conditions. Thirty-five Spanish-English BPWA and twenty-two Spanish-English healthy bilinguals completed a language use questionnaire, tests of language processing, and two verbal fluency tasks. The semantic category generation task included four conditions: two single-language conditions (No-Switch L1 and No-Switch L2) that required word production in each language separately; one dual-language condition that allowed switching between languages as desired (Self-Switch); and one dual-language condition that required switching between languages after each response (Forced-Switch). The letter fluency task required word production in single-language contexts. Overall, healthy bilinguals outperformed BPWA across all measures. Results indicate that switching is more sensitive to increased control demands than clustering, with this effect being more pronounced in BPWA, underscoring the interaction between semantic executive processes and language control in this group. Additionally, for BPWA switching performance relies on a combination of language abilities and language experience metrics.

## INTRODUCTION

Understanding the relationship between different levels of control during language production in bilingual speakers remains a central issue in the field of bilingualism. Lexical retrieval in healthy bilingual speakers may require a combination of language control processes as well as semantic executive control processes to manage competition from the unintended language as well as competing lexical representations. The relationship between these different levels of control is further complicated in bilingual patients with aphasia (BPWA) as impairments to the language system make it difficult to differentiate the two levels of control in this group. Verbal fluency tasks provide an interesting opportunity to differentiate these two levels of control in both healthy bilinguals and BPWA by examining quantitative and qualitative performance across tasks with different language demands.

When a speaker wants to produce a word in one language (e.g., *dog*), semantically related representations are also activated (e.g., *cat*) via spreading activation. Once the lexical nodes are activated, activation spreads downward to the corresponding phonological units, which in turn send activation upward to phonologically related lexical representations (e.g., the lexical node *dog* sends activation to its corresponding phonological units /d-o-g/, which in turn activate the phonologically related node *dot*) (Collins & Loftus, 1975; Dell, 1986; Dell & O’Seaghdha, 1992). This mechanism requires semantic executive control processes to manage activation of concepts and inhibit unintended nodes. These semantic executive control processes direct and control lexical activation in a contextually appropriate manner (Jefferies et al., 2008). For bilinguals, semantic representations activate lexical nodes in both languages in parallel even when only one language is in use (e.g., *dog* activates *cat* in English, as well as *perro* and *gato* in Spanish) (Bialystok et al., 2009; Colomé, 2001; Costa et al., 2000; Kroll et al., 2006), a phenomenon which is modulated by relative proficiency in each language (Dijkstra & van Heuven, 1998, 2002; Dijkstra et al., 2019; Kroll & Stewart, 1994). Since both languages are simultaneously activated, inhibitory control mechanisms are required to manage competition from the non-target language for successful language production (Green, 1998; Green & Abutalebi, 2013). Therefore, in bilingual speakers, control mechanisms are required at two levels, first at the level of language task schemas, which designate the language of intended use, and second, at the level of lexical competitors, which aid in selection of the appropriate lexical node.

More recent theories propose that different linguistic contexts require varying amounts of cognitive processes, including inhibition, goal maintenance, conflict monitoring, and interference suppression for successful language use (Green & Abutalebi, 2013). The Adaptive Control Hypothesis (ACH; Green & Abutalebi, 2013) outlines three interactional contexts for bilingual speakers, which consist of single-language, dual-language, and dense code-switching environments. In single-language contexts, speakers use one language exclusively, which requires continuous inhibition of the non-target language in order to avoid cross-language intrusions. In dual-language contexts, bilinguals switch between their two languages in a constrained manner (i.e., in responses to external environmental language cues), typically when communicating with different speakers in the same environment. This context, therefore, requires increased control processes to inhibit competitors from the non-target language. Conversely, in dense code-switching environments, bilinguals switch between their two languages as desired (i.e., in the absence of external environmental language constraints), requiring more opportunistic planning rather than direct inhibition of the non-intended language. In this context, speakers opportunistically use joint language activation, which allows them to make use of alternative forms of expression in whichever language is most readily available. One main assumption of the ACH is that in the single-language and dual-language contexts, the two languages are in a competitive relationship, while in the dense code-switching context the two languages are in a cooperative one (Green & Abutalebi, 2013). Because of this, the dual-language context is thought to place the highest cognitive control demands on language processing, whereas the dense code-switching context is thought to place the least. Additionally, for unbalanced bilinguals, higher levels of activation in the dominant language, leads to increased inhibitory demands when speaking in the less-dominant single-language context (Green, 1998).

Within the context of verbal fluency tasks, which has been extensively studied in bilingual individuals (Friesen et al., 2015; Kiran et al., 2014; Luo et al., 2010; Patra et al., 2020a; Roberts & Le Dorze, 1998) and is the focus of the current study, the ACH provides a framework to predict the influence of interactional contexts on the degree of control mechanisms required to successfully complete the task (Carpenter et al., 2020; Jevtovic et al., 2020). Within this framework, we can examine two levels of control in bilingual speakers. The first is language control, which manages competition from language task schemas via inhibitory control mechanisms for appropriate production of the intended language within the parameters of different interactional contexts. This may be examined by imposing different language demands across conditions (e.g., requiring responses only in one language vs. requiring switching between languages for each response). The second is semantic executive control, which directs activation and inhibition of lexical and phonological representations at the lexical level and may be examined by analyzing qualitative aspects of verbal fluency performance (e.g., switching and clustering performance).

**Figure 1.**
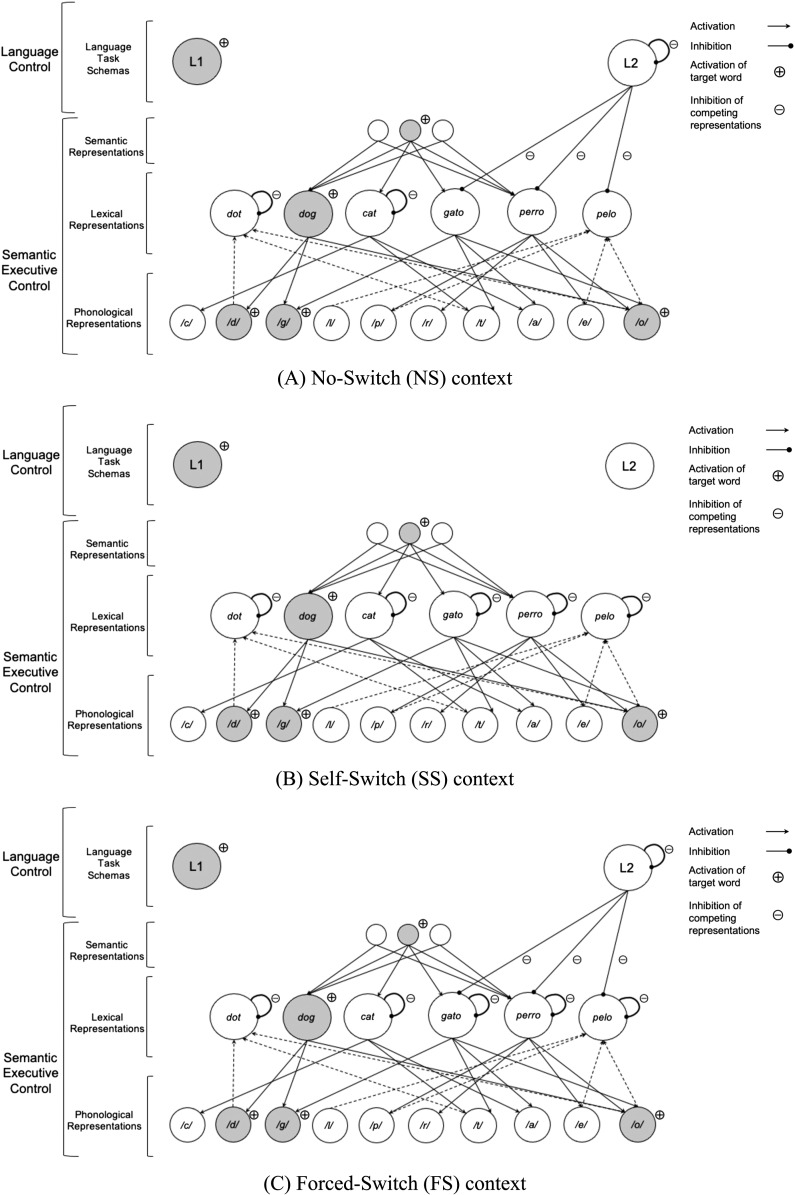
(A) Language control and semantic executive control schema for bilingual speakers in the No-Switch (NS) context, where speakers use one language exclusively. Inhibition is represented with subtraction signs. Automatic activation is represented with arrows; forward activation is shown using solid lines, and backward activation is shown using dashed lines. Activation for the intended word, *dog*, is represented with addition signs. (B) Language control and semantic executive control schema in the Self-Switch (SS) context where bilingual speakers switch between languages in an unconstrained manner (i.e., in the absence of external environmental language cues), which allows speakers to switch between languages as desired. Inhibition is represented with subtraction signs. Automatic activation is represented with arrows; forward activation is shown using solid lines, and backward activation is shown using dashed lines. Activation for the intended word, *dog*, is represented with addition signs. (C) Language control and semantic executive control schema in the Forced-Switch (FS) context, where bilingual speakers switch between languages in a constrained manner (i.e., in response to external environmental cues). Inhibition is represented with subtraction signs. Automatic activation is represented with arrows; forward activation is shown using solid lines, and backward activation is shown using dashed lines. Activation for the intended word, *dog*, is represented with addition signs.

Figure 1 aims to differentiate the control mechanisms required at the language control and semantic executive control levels in the three interactional contexts outlined by the ACH (Green & Abutalebi, 2013) as they apply to verbal fluency tasks described in this article. First, when healthy bilinguals produce items in the single-language context (represented by the No-Switch context in Figure 1A), inhibition originates from the level of the language task schemas, where activated concepts in the unintended language (in this case L2) are inhibited at the language control level rather than at the semantic executive control level. In this case, semantic executive control is required to inhibit the co-activated L1 lexical representations only. When subjects are allowed to freely switch between languages for consecutive items, as in the dense code-switching context, speakers make use of whatever lexical nodes are most readily available to facilitate lexical access through spreading activation (e.g., *dog* and possibly *gato*). In this instance, there are no language constraints, and thus, no inhibitory processes are imposed at the language level, instead, inhibitory mechanisms at the semantic executive control level are required to inhibit non-intended lexical nodes from both languages (see the Self-Switch context in Figure 1B). Finally, in the dual-language context (represented by the Forced-Switch context in Figure 1C), which requires controlled switching between target languages (e.g., *dog* and then *gato*), inhibitory mechanisms are required from both the language control and semantic executive control levels. In this case, language control is required to inhibit the previously activated L2 language task schema, and since representations at the lexical level were previously activated to produce an item in the L2, semantic executive control is required to inhibit these activated representations. This results in high cognitive demands on language control and semantic executive control mechanisms. Consequently, semantic processing may become more effortful as cognitive processes are recruited to successfully inhibit the non-target language while simultaneously managing competition from lexical representations.

**Figure 2.**
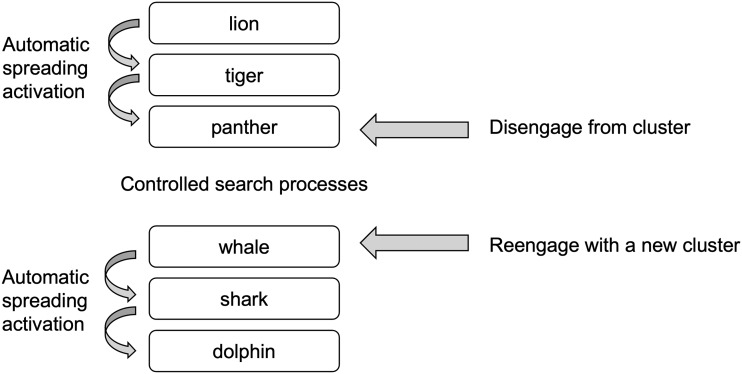
Schema of clustering and switching processes during verbal fluency tasks. This figure represents an example of clustering and switching processes during a semantic category generation task using the category “animals.” The first cluster consists of the subcategory “felines” where the speaker first produces the subcategory exemplar *lion*; in turn related concepts are then activated via automatic spreading activation, which leads the speaker to produce the exemplars *tiger* and *panther*. Once this subcategory is exhausted, the speaker must disengage from that cluster, implement controlled search processes to find another item to produce, and reengage with a new cluster, where the process begins again.

The relationship between these different control levels in bilingual speakers remains a central issue in the field of bilingualism; however, this issue is further complicated in BPWA, in whom language and cognitive control deficits are common following focal brain damage (Gray & Kiran, 2016; Helm-Estabrooks, 2002). To better understand the complex relationship between language control and semantic executive control, especially in BPWA, we assert that analyzing switching and clustering performance during verbal fluency tasks provides a means for teasing apart these two levels of control. Clustering is measured as the number of lexical items produced within a given semantic subcategory in semantic category generation and successively producing words with overlapping phonemic properties in letter fluency. Switching is a change from one semantic subcategory or phonemic cluster to another during a verbal fluency task (Figure 2). Clustering reflects the relatively automatic processes of spreading activation to related concepts from an activated concept within a given subcategory (Hughes & Bryan, 2002; Troyer et al., 1997; Unsworth et al., 2011), and is therefore associated more with the lexical component of verbal fluency tasks as it relies upon verbal memory and accessing and using the word store (Troyer et al., 1997). Conversely, switching is the ability to generate new clusters. This measure reflects strategic and controlled shifts between subcategories, and as a result, is thought to measure, to a greater extent, semantic executive control abilities (Gruenewald & Lockhead, 1980; Hughes & Bryan, 2002; Raboutet et al., 2010; Rosen et al., 2005; Unsworth et al., 2011). Studies have shown that effective performance on verbal fluency tasks requires a balance between clustering and switching processes (Troyer et al., 1997).

Previous studies investigating switching and clustering performance in patients with aphasia (PWA) have shown that monolingual PWA produce fewer correct words, smaller cluster sizes, and fewer switches than healthy monolinguals (Bose et al., 2017), reflecting difficulties in both lexical (fewer words and smaller clusters) and executive (fewer switches) components of this task. Comparably, Kiran and colleagues (2014) found that BPWA produce reduced number of words, smaller mean semantic cluster sizes, and switch fewer times across both languages compared to healthy bilinguals. However, BPWA showed a dissociation between switching and clustering performance (e.g., switching was more impaired than clustering), indicating that BPWA may show different degrees of impairment in automatic versus controlled search mechanisms (Kiran et al., 2014). These studies highlight the need for research to investigate the interaction between these two variables and how they may be differentially impacted with changing language demands of the task.

Recent studies have used a fluency difference score (FDS) as a measure of cognitive control ability in verbal fluency tasks (Friesen et al., 2015; Patra et al., 2020a). FDS is calculated by taking the difference in performance between the category generation and letter fluency tasks as a proportion of correct responses on the category generation task. Given that letter fluency is thought to have higher cognitive control demands while semantic category generation is thought to place greater emphasis on lexico-semantic abilities (Luo et al., 2010; Patra et al., 2020b; Shao et al., 2014), a smaller FDS is thought to be indicative of better cognitive control abilities (Friesen et al., 2015; Patra et al., 2020b). A recent study by Patra et al. (2020b) found that BPWA produced fewer correct words, had larger FDSs, and switched fewer times as compared to healthy bilinguals; however, both groups demonstrated similar cluster scores. Additionally, they found that BPWA with better inhibitory control produced more correct words and a greater number of semantic switches, further supporting the role of cognitive control in switching performance.

To expand these early and interesting results, the present study examines switching and clustering strategies in healthy bilinguals and BPWA with the goal of disentangling the potential interactions among language proficiency, bilingual language and semantic executive control, and focal brain damage. Previous work has examined the influence of varying cognitive control demands in semantic category generation performance in both healthy bilinguals (Jevtovic et al., 2020) and BPWA (Carpenter et al., 2020). These studies implemented four language conditions (two single-language conditions and two dual-language conditions), as a way of varying the cognitive control demands of the task. The single-language conditions consisted of two No-Switch (NS-L1 and NS-L2) conditions, where participants produced lexical items within a given semantic category using one language only (Figure 1A). The first dual-language condition was the Self-Switch (SS) condition, where participants switched between languages as desired (Figure 1B). Finally, the second dual-language condition was the Forced-Switch (FS) condition, in which participants were required to switch languages after each response (Figure 1C). Both studies found superior performance in the SS and NS-L1 conditions compared to the NS-L2 and FS conditions for the healthy bilinguals, and Carpenter and colleagues (2020) additionally found that BPWA demonstrated comparable performance to healthy bilinguals only in the SS condition. These results highlight that BPWA were more sensitive to the impact of cognitive control on lexical retrieval in the conditions where the two languages are in a competitive relationship (i.e., NS-L1, NS-L2, and FS conditions) but not in the condition where the two languages are in a cooperative relationship (i.e., SS condition), suggesting that increased control demands arising from the level of language control may hinder communicative effectiveness in BPWA.

Therefore, in the current study, we examined verbal fluency switching and clustering performance within four different language contexts (NS-L1, NS-L2, SS, and FS) as a method for teasing apart language and semantic executive control processes in BPWA and healthy bilinguals. The following research questions were posed.

(i)  *How do cognitive control abilities differ between L1 and L2?* First, we hypothesized that healthy bilinguals would demonstrate smaller FDSs than BPWA, reflective of control impairments following acquired brain injury (ABI). Furthermore, it was expected that FDSs would be larger in L2 compared to L1, indicating greater control abilities for both groups in L1.
(ii)  *How does switching and clustering performance differ for BPWA and healthy bilinguals across the different conditions of the semantic category generation task?* Given the findings of previous research (Kiran et al., 2014; Patra et al., 2020a), it was hypothesized that healthy bilinguals would produce larger mean semantic cluster sizes and a greater number of switches compared to BPWA, reflective of varying degrees of language impairment in the BPWA group. When looking at performance across conditions, we anticipated that clustering performance would be comparable across the four conditions for both groups as it is a measure of automatic spreading activation and should therefore be minimally impacted by increasing language control demands. Conversely, we expected that in the conditions where both languages are in a competitive relationship (i.e., in the NS-L1, NS-L2, and FS conditions), switching performance would be compromised for BPWA, reflective of competing control processes between language task schemas and semantic executive control processes.
(iii)  *How does switching and clustering performance differ for BPWA and healthy bilinguals across the different conditions of the letter fluency task?* Like the semantic category generation task, it was hypothesized that healthy bilinguals would outperform BPWA on both switching and clustering measures. Additionally, it was expected that, while healthy bilinguals would perform better in LF-L1, BPWA would demonstrate reduced performance in both languages, reflective of the increased semantic executive control demands imposed by this task, as semantic competition during phonological searching requires more controlled search processes rather than reliance on automatic activation of related concepts.
(iv)  *What factors predict clustering and switching performance in the SS and FS conditions?* It was hypothesized that there would be a dissociation between measures that contributed to switching and clustering performance for both groups. Specifically, standardized assessment scores including scores on the Pyramids and Palm Trees test (PAPT; Howard & Patterson, 1992) and selected subtests of the Bilingual Aphasia Test (BAT; Paradis, 1989) would better predict clustering performance in both conditions, as higher activation of lexical representations would lend itself to better ability to extend semantic clusters. Additionally, it was hypothesized that FS switching performance would be predicted by language experience measures (language use questionnaire, or LUQ, metrics) as increased proficiency would lead to greater efficiency at implementing language control, resulting in less competition between the language control and semantic executive control levels. Finally, for BPWA, it was hypothesized that Raven’s Coloured Progressive Matrices (RCPM; Kertesz, 2006) scores, a measure of matrix reasoning (Fong et al., 2020), would be predictive of performance on FS switching performance as better nonverbal cognitive function would lead to more controlled search processes as task demands increase.

## MATERIALS AND METHODS

### Participants

Thirty-five Spanish-English BPWA (19 females; mean age = 52.9, *SD* = 16.4; mean education = 14.9, *SD* = 2.7; mean age of acquisition (AoA) of the second language (L2) = 10.5, *SD* = 7.9) and 22 age-matched Spanish–English healthy bilinguals (19 females; mean age = 47.2; *SD* = 15.4) participated in this study [*t*(1, 55) = −1.3268, *p* = 0.19]. While the groups were matched for age, there were between group differences in education (healthy bilinguals mean = 17.6, *SD* = 5.2) [*t*(1, 51) = 2.5041, *p* = 0.016]) and L2 AoA (healthy bilinguals mean = 17.0, *SD* = 11.9) [*t*(1, 52) = 2.4968, *p* = 0.016]). Nonetheless, 25 BPWA and 21 healthy bilinguals reported Spanish as their first-acquired language (L1), so both groups consisted of mainly L1 Spanish speakers. All BPWA were at least 6 months post-onset (mean MPO = 61.0, *SD* = 86.2) and presented with aphasia secondary to stroke (*n* = 33), traumatic brain injury (*n* = 1), or tumor (*n* = 1). All participants gave their written informed consent.

Participants completed an LUQ (Kastenbaum et al., 2019), which captured information regarding each participant’s language use, exposure, and self-rated proficiency for each language separately over their lifetime. Specifically, the LUQ obtained information regarding L2 AoA; language usage on weekdays and weekends measured on an hourly basis; lifetime exposure for hearing, speaking, and reading in each language; lifetime confidence for hearing, speaking, and reading in each language; family proficiency for parents and siblings; educational history regarding language use and preferences of the participant and peers during elementary school, high school, and college; and language ability rating of each participant’s self-rated abilities in speaking, listening, reading, and writing for each language. Language usage and language ability ratings in each language were collected both pre- and post-ABI for the BPWA to reflect changes in these metrics after aphasia onset. For BPWA, caregivers were present to corroborate LUQ metrics including patients’ language use patterns pre- and post-ABI, language abilities ratings, and exposure in each language. Table 1 summarizes the LUQ information of the BPWA and the healthy bilinguals. Independent samples *t* tests revealed no differences in their levels of relative language experience. Table 2 summarizes lesion information and aphasia subtypes of the BPWA.

**Table 1.** Average percentage of the Language Use Questionnaire metrics for bilingual patients with aphasia and healthy bilinguals.

**Group**		**Pre-ABI LAR**		**Post-ABI LAR**		**Pre-ABI language use**		**Post-ABI language use**		**Lifetime exposure**		**Lifetime confidence**		**Family proficiency**		**Educational history**	
		**L1**	**L2**	**L1**	**L2**	**L1**	**L2**	**L1**	**L2**	**L1**	**L2**	**L1**	**L2**	**L1**	**L2**	**L1**	**L2**
BPWA	*n*	32	32	30	30	34	34	32	32	34	34	34	34	34	34	34	34
	Mean (%)	93.5	81.6	59.5	49.7	51.6	48.4	65.0	35.1	57.6	42.4	91.7	61.7	94.5	58.6	72.2	27.8
	*SD*	11.3	17.2	21.3	20.2	30.0	30.0	27.0	27.0	20.4	20.4	13.6	23.4	12.3	32.6	26.9	26.9
HB	*n*	22	22	N/A	N/A	22	22	N/A	N/A	22	22	22	22	21	21	22	22
	Mean (%)	98.0	83.1	N/A	N/A	45.5	54.5	N/A	N/A	66.6	33.4	97.6	49.5	96.9	43.7	81.5	18.5
	*SD*	5.9	16.4	N/A	N/A	33.4	33.4	N/A	N/A	18.8	18.8	5.5	27.5	12.7	25.8	25.2	25.2
*p* value		0.091	0.742	N/A	N/A	0.292	0.291	N/A	N/A	0.104	0.104	0.059	0.086	0.509	0.085	0.207	0.085

*Note*. LAR = language ability rating; BPWA = bilingual patients with aphasia; HB = healthy bilingual; ABI = acquired brain injury; N/A = no data available.

**Table 2.** Lesion and clinical information for bilingual patients with aphasia.

**Code**	**Age**	**Sex**	**MPO**	**L1**	**Lesion information**	**L1 WAB AQ**	**L1 Aphasia subtype**	**L2 WAB AQ**	**L2 Aphasia subtype**
BPWA1	82	M	411	Span.	Right posterior parieto-occipital infarct	55.7	Broca’s	29.6	Global
BPWA2	54	F	52	Span.	Left border zone infarcts between the ACA and MCA	74.1	Anomic	68.5	Broca’s
BPWA3	25	F	18	Eng.	Multiple CVAs secondary to Moyamoya disease	93.4	Anomic	81.4	Anomic
BPWA4	44	M	20	Eng.	Left MCA CVA	89.8	Anomic	84.5	Anomic
BPWA5	63	F	28	Span.	Left CVA	N/A	N/A	20.7	Global
BPWA6	24	F	6	Span.	Left CVA	27.3	Broca’s	37.3	Broca’s
BPWA7	26	F	130	Span.	Left tumor in posterior centrum semiovale	77.5	Anomic	67.6	Wernicke’s
BPWA8	58	F	70	Span.	Left CVA	N/A	N/A	N/A	N/A
BPWA9	48	M	53	Span.	TBI	N/A	N/A	N/A	N/A
BPWA10	66	M	339	Span.	Left MCA CVA	N/A	N/A	N/A	N/A
BPWA11	47	F	53	Span.	Left MCA temporoparietal infarct	79.1	Conduction	54.4	Broca’s
BPWA12	53	M	38	Span.	Left CVA	51.3	Wernicke’s	47.5	Wernicke’s
BPWA13	56	F	104	Eng.	Left frontoparietal hemorrhage	98.2	Anomic	N/A	N/A
BPWA14	77	M	27	Span.	Left MCA CVA involving precentral gyrus	67.4	Broca’s	64.7	Broca’s
BPWA15	78	F	40	Span.	Left MCA hemorrhage	78.9	Anomic	76.8	Conduction
BPWA16	70	M	10	Span.	Left frontal lobe CVA	57.3	Conduction	39.8	Broca’s
BPWA17	27	F	56	Span.	Left CVA	72.3	Anomic	66.4	Broca’s
BPWA18	53	F	51	Eng.	Left MCA and PCA CVAs	90	Anomic	68.8	Conduction
BPWA19	37	F	10	Span.	Left CVA	N/A	N/A	69.8	Conduction
BPWA20	54	M	6	Span.	Subacute left MCA CVA	N/A	N/A	N/A	N/A
BPWA21	69	M	16	Eng.	Left CVA	35.9	Wernicke’s	46.5	Conduction
BPWA22	54	F	29	Eng.	Left CVA	96.5	Anomic	60.8	Broca’s
BPWA23	47	F	16	Span.	Left anterior MCA infarct	82.4	Anomic	71.2	Conduction
BPWA24	46	M	12	Span.	TIA	N/A	N/A	N/A	N/A
BPWA25	56	M	54	Span.	Left CVA	81.2	Anomic	91	Anomic
BPWA26	39	M	42	Span.	Left CVA	21	Broca’s	39.5	Broca’s
BPWA27	42	M	24	Eng.	Left CVA	94.6	Anomic	57.8	Broca’s
BPWA28	62	M	54	Eng.	Left CVA	89.2	Anomic	78.6	Anomic
BPWA29	81	F	164	Span.	Left CVA, right occipital and right cerebellar infarcts	N/A	N/A	N/A	N/A
BPWA30	21	F	23	Span.	Left CVA	34.4	Broca’s	53.3	Wernicke’s
BPWA31	64	M	6	Eng.	Left CVA, Hx of right CVA	47.7	Broca’s	16.4	Broca’s
BPWA32	66	F	11	Span.	Left MCA CVA	83	Conduction	70.4	Conduction
BPWA33	56	M	47	Span.	Left CVA	92.6	Anomic	97.2	Anomic
BPWA34	65	F	78	Span.	Left MCA CVA	N/A	N/A	N/A	N/A
BPWA35	44	F	36	Eng.	Left MCA CVA	N/A	N/A	N/A	N/A

*Note*. Aphasia subtypes are based on WAB-R (Kertesz, 2006) classifications; for more detailed information regarding classification of aphasia subtypes refer to the WAB-R scoring manual. MPO = months post-onset; L1 = first-acquired language; Span. = Spanish; Eng. = English; ACA = anterior cerebral artery; MCA = middle cerebral artery; CVA = cerebrovascular accident; TBI = traumatic brain injury; Hx = prior medical history; PCA = posterior cerebral artery; TIA = transient ischemic attack; WAB = Western Aphasia Battery; AQ = Aphasia Quotient; N/A = no data available; L2 = second-acquired language.

### Standardized Assessments

Participants also completed additional language testing in English and Spanish that included (a) a 60-item naming screener consisting of high frequency and concrete items (Peñaloza, Grasemann, et al., 2019) and (b) the Boston Naming Test (BNT; Kaplan et al., 2001; Kohnert et al., 1998) to assess picture naming abilities; (c) selected subtests of the Psycholinguistic Assessment of Language Processing in Aphasia (PALPA; Kay et al., 1992) and its Spanish translation (EPLA; Kay et al., 1995) including PALPA 47/ EPLA 45, PALPA 48/ EPLA 46, PALPA 49/ EPLA 47 and PALPA 50/EPLA 48 to assess lexico-semantic processing; (d) four subtests of the BAT (Paradis, 1989) including semantic categories, synonyms, antonyms I, and antonyms II to assess semantic processing; and (e) the PAPT test (Howard & Patterson, 1992) to assess nonverbal semantic knowledge. Additionally, matrix reasoning was evaluated only in the BPWA using the RCPM subtest from the Western Aphasia Battery–Revised (WAB-R; Kertesz, 2006). A summary of performance on standardized measures for BPWA and healthy bilinguals is included in Table 3.

**Table 3.** Average percentage of correct responses on standardized measures for bilingual patients with aphasia and healthy bilinguals.

**Group**		**Verbal assessments**								**Nonverbal assessments**	
		**Naming screener**		**BNT**		**PALPA/EPLA composite**		**BAT composite**			
		**L1**	**L2**	**L1**	**L2**	**L1**	**L2**	**L1**	**L2**	**PAPT**	**RCPM**
BPWA	*n*	31	31	29	32	23	25	29	28	29	25
	Mean (%)	56.5	42.7	44.1	32.8	82.6	74.8	69.1	64.6	89.2	74.5
	*SD*	29.1	29.0	26.2	24.0	10.0	13.2	26.7	20.3	10.7	16.9
HB	*n*	22	22	22	22	22	22	22	22	22	N/A
	Mean (%)	84.6	78.6	76.8	62.8	93.2	87.5	92.7	83.5	94.4	N/A
	*SD*	9.6	15.5	15.2	18.0	6.9	8.1	11.0	17.0	5.7	N/A
*p* value		<0.001	<0.001	<0.001	<0.001	<0.001	<0.001	<0.001	<0.001	0.044	N/A

*Note*. BPWA = Bilingual patients with aphasia; HB = healthy bilingual; BNT = Boston Naming Test; PALPA/EPLA = Psycholinguistic Assessment of Language Processing in Aphasia in English and Spanish respectively; BAT = Bilingual Aphasia Test; PAPT = Pyramids and Palm Trees; RCPM = Raven’s Coloured Progressive Matrices; L1 = first-acquired language; L2 = second-acquired language; N/A = no data available.

### Experimental Tasks

Two verbal fluency tasks were administered: a modified semantic category generation task (i.e., producing items in a given semantic category in 60 s) and a traditional letter fluency task (i.e., producing items beginning with a given letter in 60 s in both languages). In the category generation task (Carpenter et al., 2020), participants were to produce words in four semantic categories: animals, clothing, food, and modes of transportation. The conditions for the category generation task consisted of (a) two No-Switch conditions (NS-L1 and NS-L2) where participants produced responses in one language exclusively, (b) one Self-Switch condition (SS) where participants could switch between languages as desired, and (c) one Forced-Switch condition (FS) where participants were required to switch between languages after each response. Each condition was administered twice across two separate days. On day one, instructions were administered only in L1 and tasks NS-L1, NS-L1, SS, and FS were administered, and on day two all instructions were provided in L2, and tasks NS-L2, NS-L2, SS, and FS were administered. The category-to-condition assignments were counterbalanced across participants to account for the potential impact of semantic category knowledge on condition performance. In the letter fluency task, participants were to produce words beginning with the letters “F,” “A,” and “S” in English (Controlled Oral Word Association Test; Benton & Hamsher, 1976) and “P,” “M,” and “R” in Spanish (Peña-Casanova et al., 2009), as these letter combinations are frequently administered and show similar norms across English and Spanish respectively. The order of administration for the letter fluency task was counterbalanced across participants. Both tasks have been described in detail elsewhere (Carpenter et al., 2020).

### Data Coding and Scoring Procedures

All data including bilingual language history metrics, performance on standardized language assessments, and performance on the semantic category generation task were coded as being produced in each participant’s first-acquired L1 or second-acquired L2 as self-reported in their LUQ. Responses in the verbal fluency tasks were recorded in audio and in written form during the testing session and verified for accuracy afterwards via the audio recording. In the semantic category generation task, since each condition was administered twice, scores were created for each condition by averaging the total number of correct words across the two administrations. Responses were correct if they were unique words in the target category, produced in the target language, were not a repetition of a previously produced response, and contained no more than one phonemic substitution, omission, or addition.

Fluency difference scores were computed as the difference in the number of correct responses between category generation and letter fluency tasks as a proportion of correct responses in the category generation task, to reflect the role of cognitive control in verbal fluency tasks (Friesen et al., 2015; Patra et al., 2020b). FDSs were computed in each language by using the number of correct responses in the NS and LF conditions in L1 and L2 separately.

Switching and clustering analyses closely followed the methods outlined by Troyer et al. (1997), where semantic clustering was defined as successively producing words that shared a semantic subcategory (e.g., *dog*, *cat* (pets) or *horse*, *pig*, *cow* (farm animals)), and phonemic clustering was defined as successively generating words that fulfilled any one of the following criteria: (i) words that began with the same first two sounds (e.g., *art*, *arm*), (ii) words that differed only by a vowel sound regardless of the actual spelling (e.g., *fat*, *fit*, *foot*), (iii) words that rhymed (e.g., *sand*, *stand*), or (iv) words that were homonyms (e.g., *sum*, *some*). Cluster size was calculated starting with the second word in each cluster, such that a single word (e.g., *dog*) was given a cluster size of zero, two-word clusters (e.g., *dog*, *cat*) were given a cluster size of one, and so on. Mean cluster size was calculated by adding the size of each cluster and dividing the total score by the number of clusters, such that *dog*, *cat*, *horse*, *pig*, *cow* would receive a mean cluster size of 1.5 (a cluster size of 1 for *dog*, *cat* plus a cluster size of 2 for *horse*, *pig*, *cow* averaged to get 1.5). Switches were calculated by tallying the number of transitions between clusters, including single words (e.g., *dog*, *cat*, *horse*, *pig*, *cow*, *dolphin* would contain two switches, after *cat* and after *cow*). Errors and repetitions were included in both the clustering and switching analyses.

### Statistical Analyses

First, to assess cognitive control abilities in L1 and L2, we conducted a two-way repeated measures ANOVA with Group (BPWA and HB) as the between-subject factor and Language (L1 and L2) as the within-subject factor for FDSs (research question 1). To compare the mean semantic cluster size and number of switches produced by the two groups across the four experimental conditions of the semantic category generation task (research question 2), two 2-way repeated measures ANOVAs were conducted with Group (BPWA and HB) as the between-subject factor and Condition (SS, NS-L1, NS-L2, and FS) as the within-subject factor for mean semantic cluster size and number of semantic switches separately. Similarly, for research question 3, similar analyses were conducted with Group as the between-subject and Condition (LF-L1 and LF-L2) as the within-subject factor for both mean phonemic cluster size and number of phonemic switches. Additionally, since different between-group effects were expected for different conditions, planned paired-samples *t* tests were conducted across conditions for each group separately, even if no significant interaction effect was observed. Furthermore, to examine a main effect of Group, planned independent samples *t* tests were conducted to investigate specific differences between groups in each condition. All *p* values were corrected for multiple comparisons using the FDR approach.

To determine which pre- and post-ABI LUQ factors loaded onto different components (research question 4), principal component analyses (PCAs) were first conducted for BPWA and healthy bilinguals separately as done in previous research (Kastenbaum et al., 2019; Peñaloza, Barrett, & Kiran, 2019). These PCAs were performed for L1 and L2 separately for a larger sample of BPWA (*n* = 59) and healthy bilinguals (*n* = 31) that overlapped with this project (Marte et al., 2021). (Results of the PCA for the entire sample of BPWA can be found at https://www.bu.edu/aphasiaresearch/resources/principal-component-analysis-of-bilinguals-with-aphasia/ and for healthy bilinguals at https://www.bu.edu/aphasiaresearch/resources/principal-component-analysis-of-luq-metrics-in-bilingual-healthy-controls/.) Once these PCAs were performed, individual factor scores were extracted for the present cohort of BPWA (*n* = 32) and healthy bilinguals (*n* = 22) for whom data were available. A varimax normalized factor rotation was used to examine the factor loadings of components with factor loadings >0.6, and the scores were extracted using the default regression function from the psych package in R (https://cran.r-project.org/web/packages/psych/index.html). Individual factor scores were then included in four linear regression models along with language scores (L1 BAT, L2 BAT, and PAPT) for both groups and RCPM scores for BPWA only, to determine which factors predicted performance on SS average cluster size, SS number of switches, FS average cluster size, and FS number of switches. The regression analyses were only conducted in the dual-language conditions, as we were interested in teasing apart the impact of language factors that contributed to performance in the condition with the least demands of cognitive control (e.g., SS) compared to the condition with the highest demands of cognitive control (e.g., FS). Backward stepwise regressions were conducted to determine which factors best contributed to the models. These four linear regressions were conducted for BPWA and healthy bilinguals separately.

## RESULTS

While the number of correct responses was not the focus of the current study and has previously been reported elsewhere for a subset of patients included in this study (Carpenter et al., 2020), we have summarized performance on the category generation and letter fluency tasks below as they provide the basis for results reported in this article (Table 4).

**Table 4.** Number of correct responses produced across the two verbal fluency tasks.

**Condition**	**BPWA (*n* = 35)**		**HB (*n* = 22)**		***p* value**	**Cohen’s *d***
	**Mean**	***SD***	**Mean**	***SD***		
SS	7.34	5.10	18.05	6.43	<0.001	1.85
NS-L1	7.63	4.52	16.68	6.51	<0.001	1.61
NS-L2	5.71	4.87	15.50	5.32	<0.001	1.92
FS	5.31	4.25	12.64	5.95	<0.001	1.42
LF-L1	4.01	3.38	13.11	3.97	<0.001	2.47
LF-L2	3.02	3.52	10.51	3.23	<0.001	2.22

*Note*. BPWA = bilingual patients with aphasia; HB = healthy bilinguals; SS = Self-Switch; NS-L1 = No Switch (L1); NS-L2 = No Switch (L2); FS = Forced-Switch; LF-L1 = Letter Fluency, L1; LF-L2 = Letter Fluency, L2. BPWA produced significantly fewer correct responses than healthy bilinguals across all conditions. Pairwise comparisons revealed superior performance in the SS condition compared to the NS-L2 (*p* = 0.004) and FS (*p* < 0.001) conditions, superior performance in the NS-L1 condition compared to the NS-L2 (*p* = 0.012) and FS conditions (*p* < 0.001), and superior performance in the NS-L2 condition compared to the FS condition (*p* = 0.019). No differences were observed between the SS and NS-L1 condition (*p* = 0.268). Additionally, participants showed superior performance in LF-L1 compared to LF-L2 (*p* < 0.001).

### Fluency Difference Score

**Figure 3.**
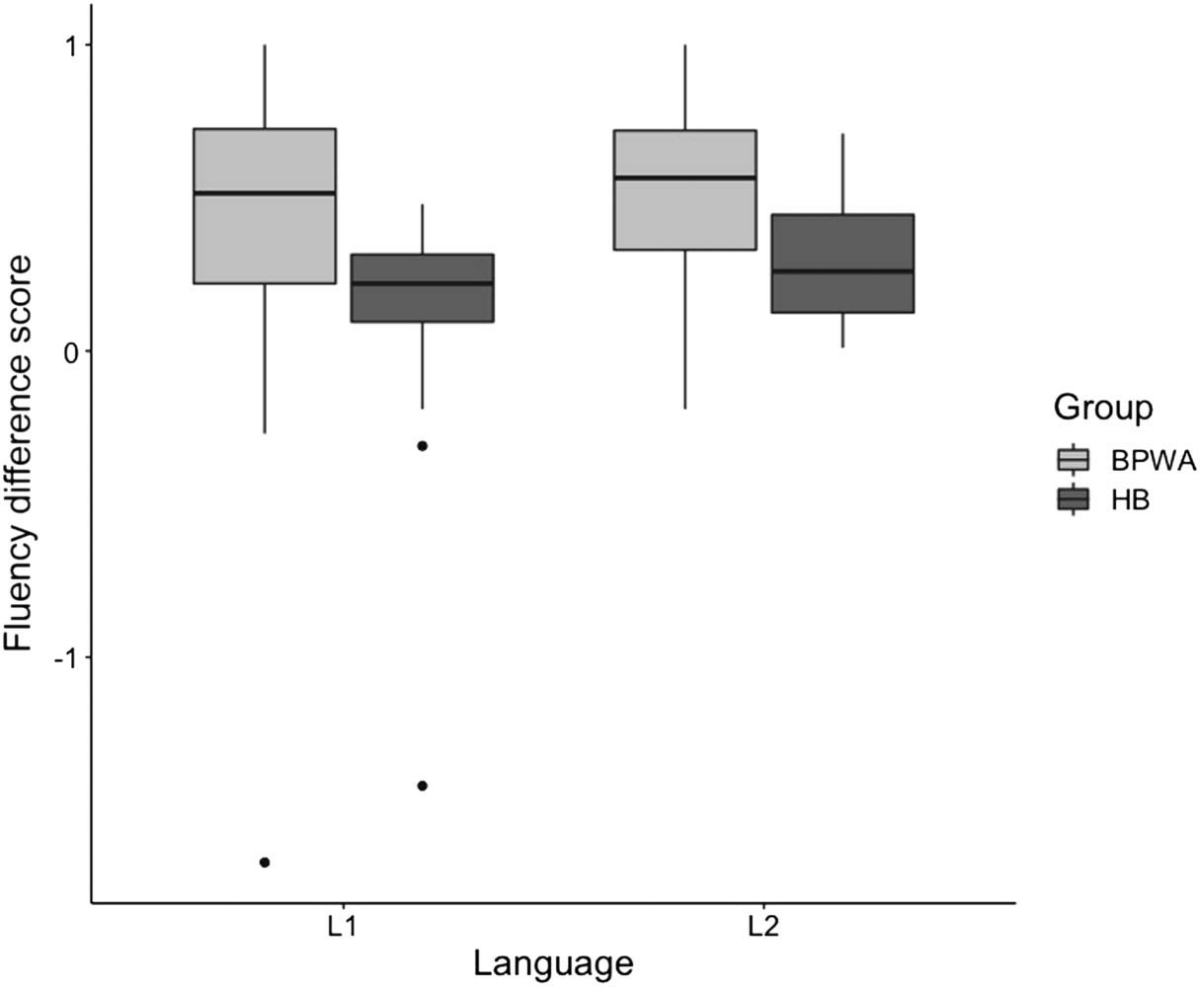
Fluency difference scores (FDSs) of the bilingual patients with aphasia (BPWA) and healthy bilinguals (HB). Boxplots are shown separately for each group across the two languages (L1 and L2). BPWA showed significantly larger FDSs relative to the HB in both languages (statistical significance at the 0.05 level).

Results from the repeated-measures ANOVA revealed a significant main effect of Group [*F*(1, 54) = 7.847, *p* = 0.007, *η*_*p*_^2^ = 0.127] and Language [*F*(1, 54) = 5.113, *p* = 0.028, *η*_*p*_^2^ = 0.087]; however, the Group × Language interaction was not significant [*F*(1, 54) = 0.356, *p* = 0.553, *η*_*p*_^2^ = 0.007], indicating that the BPWA had significantly larger FDSs than healthy bilinguals, and that overall FDSs in L1 were smaller compared to L2 (*p* = 0.002) for both groups (Figure 3). Independent samples *t* tests revealed that BPWA produced significantly larger FDSs than healthy bilinguals in L1 [*t*(54) = 2.209, *p* = 0.031, *p*-adj = 0.031, *d* = 0.618] and L2 [*t*(54) = −2.644, *p* = 0.011, *p*-adj = 0.022, *d* = 0.484].

### Semantic Clustering and Switching Analysis

#### Semantic clustering

**Figure 4.**
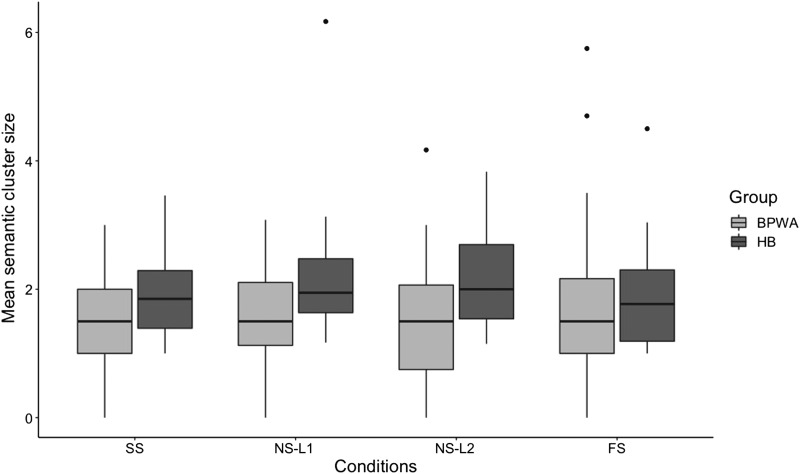
Mean semantic cluster sizes of the bilingual patients with aphasia (BPWA) and healthy bilinguals (HB) on the semantic category generation task. Boxplots are shown separately for each group across the four conditions (SS, NS-L1, NS-L2, and FS). BPWA showed significantly smaller mean semantic cluster sizes relative to the HB in all conditions except for the FS condition.

Results from the repeated-measures ANOVA revealed a significant main effect of Group [*F*(1, 55) = 6.721, *p* = 0.012, *η*_*p*_^2^ = 0.095], although the main effect of Condition [*F*(3, 53) = 0.957, *p* = 0.420, *η*_*p*_^2^ = 0.045] and the Group × Condition interaction were not significant [*F*(3, 53) = 0.616, *p* = 0.608, *η*_*p*_^2^ = 0.076], indicating that BPWA produced smaller mean semantic cluster sizes than healthy bilinguals, although groups did not demonstrate differences in performance across conditions (Figure 4). Independent samples *t* tests revealed that BPWA produced significantly smaller average semantic cluster sizes than healthy bilinguals in the SS [*t*(55) = −2.291, *p* = 0.026, *p*-adj = 0.037, *d* = 0.648], NS-L1 [*t*(55) = −2.256, *p* = 0.028, *p*-adj = 0.037, *d* = 0.591], and NS-L2 [*t*(55) = −2.491, *p* = 0.016, *p*-adj = 0.037, *d* = 0.676] conditions; however no group differences were observed in the FS condition [*t*(55) = −0.514, *p* = 0.609, *p*-adj = 0.609, *d* = 0.146], indicating comparable mean semantic cluster sizes across groups in this condition.

#### Semantic switching

**Figure 5.**
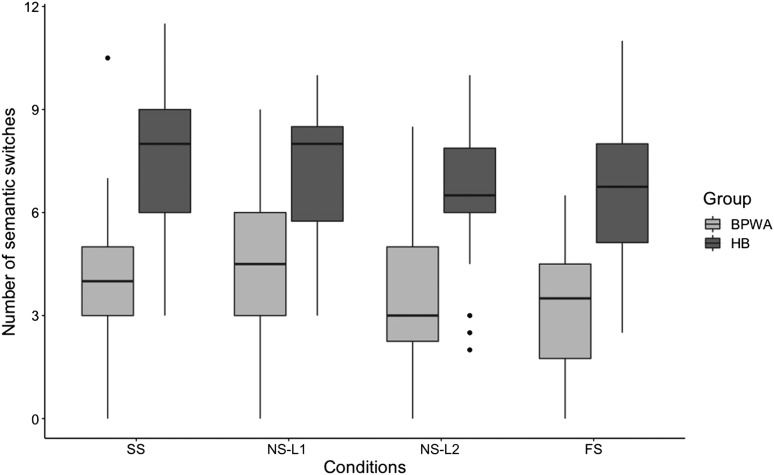
Number of semantic switches produced by the bilingual patients with aphasia (BPWA) and healthy bilinguals (HB) on the semantic category generation task. Boxplots are shown separately for each group across the four conditions (SS, NS-L1, NS-L2, and FS). BPWA produced significantly fewer semantic switches relative to the HB in all conditions.

Results from the repeated-measures ANOVA revealed a significant main effect of Group [*F*(1, 55) = 53.870, *p* < 0.001, *η*_*p*_^2^ = 0.490] and Condition [*F*(3, 53) = 6.140, *p* = 0.001, *η*_*p*_^2^ = 0.258], although the Group × Condition interaction was not significant [*F*(3, 53) = 0.966, *p* = 0.416, *η*_*p*_^2^ = 0.052], indicating that BPWA produced fewer semantic switches than healthy bilinguals and performance differed across conditions for both groups (Figure 5). Specifically, pairwise comparisons revealed significantly more switches produced in the SS compared to the NS-L2 (*p* = 0.015) and FS (*p* = 0.002) conditions, and significantly more switches produced in the NS-L1 condition compared to the NS-L2 (*p* = 0.001) and FS (*p* = 0.004) conditions. No significant differences in number of switches were observed between the SS and NS-L1 conditions and the NS-L2 and FS conditions (*p* > 0.05 in all cases). Independent samples *t* tests revealed that BPWA produced significantly fewer switches than healthy bilinguals in all conditions (*p*-adj < 0.001 in all cases). To further investigate performance across conditions within each group, paired-samples *t* tests were performed for BPWA and healthy bilinguals separately. Results revealed that BPWA produced significantly more switches in SS compared to FS [*t*(34) = 2.517, *p* = 0.017, *p-*adj = 0.034] and NS-L1 compared to both NS-L2 [*t*(34) = 3.276, *p* = 0.002, *p*-adj = 0.006] and FS [*t*(34) = 3.688, *p* = 0.001, *p*-adj.= 0.006]; however no differences arose between SS and NS-L1 [*t*(34) = 1.586, *p* = 0.112, *p*-adj = 0.168], SS and NS-L2 [*t*(34) = 1.322, *p* = 0.195, *p*-adj = 0.234], or NS-L2 and FS [*t*(34) = 0.993, *p* = 0.328, *p*-adj = 0.328]. Healthy bilinguals did not significantly differ across any conditions after correcting for multiple comparisons (*p*-adj > 0.192 in all cases).

### Phonemic Switching and Clustering Analysis

#### Phonemic clustering

**Figure 6.**
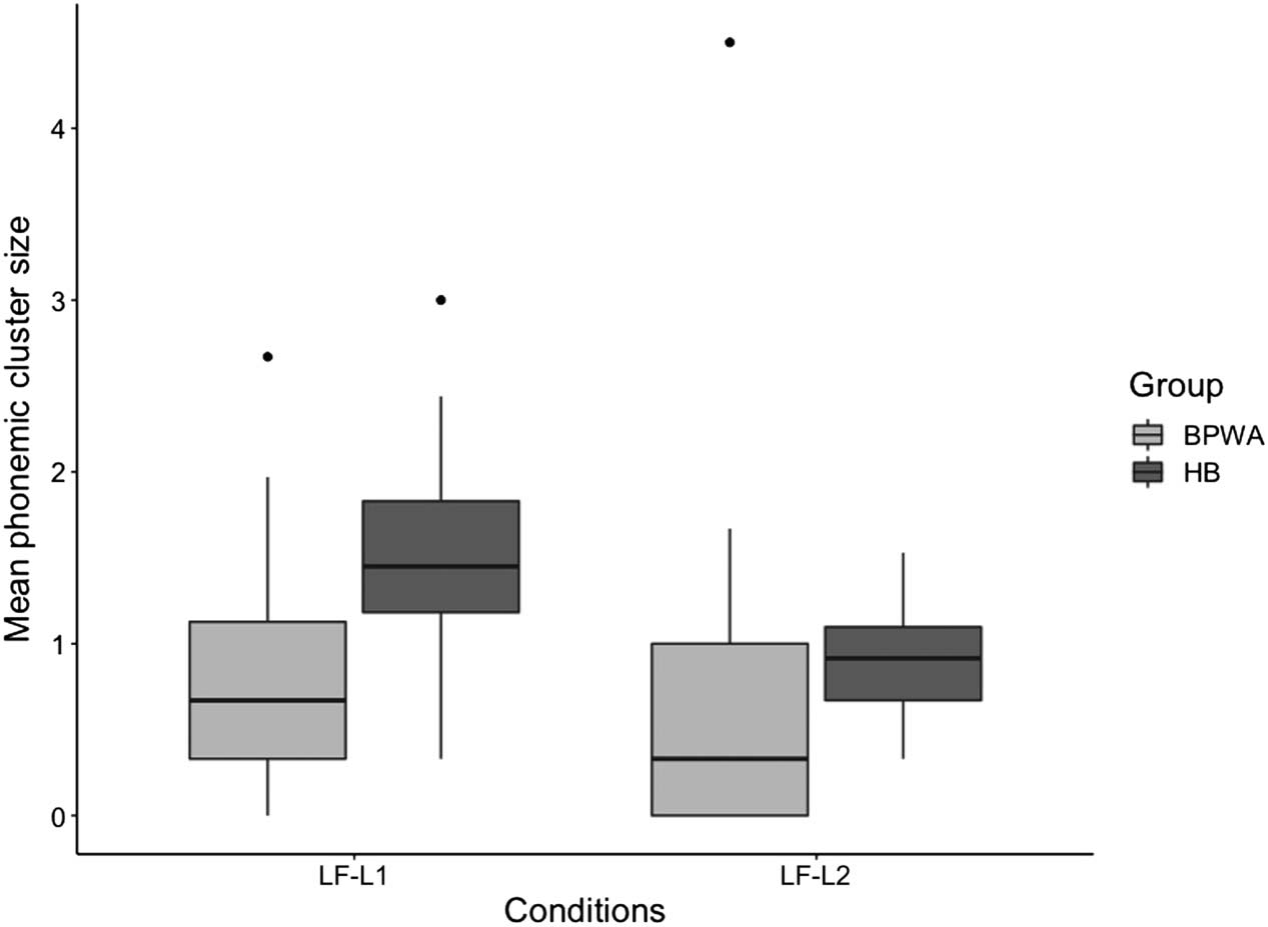
Mean phonemic cluster sizes of the bilingual patients with aphasia (BPWA) and healthy bilinguals (HB) on the letter fluency task. Boxplots are shown separately for each group across the two conditions (LF-L1 and LF-L2). BPWA produced significantly smaller mean phonemic cluster sizes relative to the HB in the LF-L1 condition but not the LF-L2 condition.

Results from the repeated-measures ANOVA revealed a significant main effect of Group [*F*(1, 54) = 11.257, *p* < 0.001, *η*_*p*_^2^ = 0.173], Condition [*F*(1, 54) = 10.560, *p* = 0.002, *η*_*p*_^2^ = 0.164], and Group × Condition interaction [*F*(1, 54) = 5.900, *p* = 0.019, *η*_*p*_^2^ = 0.098], indicating that, overall, the BPWA produced smaller mean phonemic cluster sizes than healthy bilinguals and only healthy bilinguals showed superior performance in LF-L1 compared to LF-L2 (Figure 6).

#### Phonemic switching

**Figure 7.**
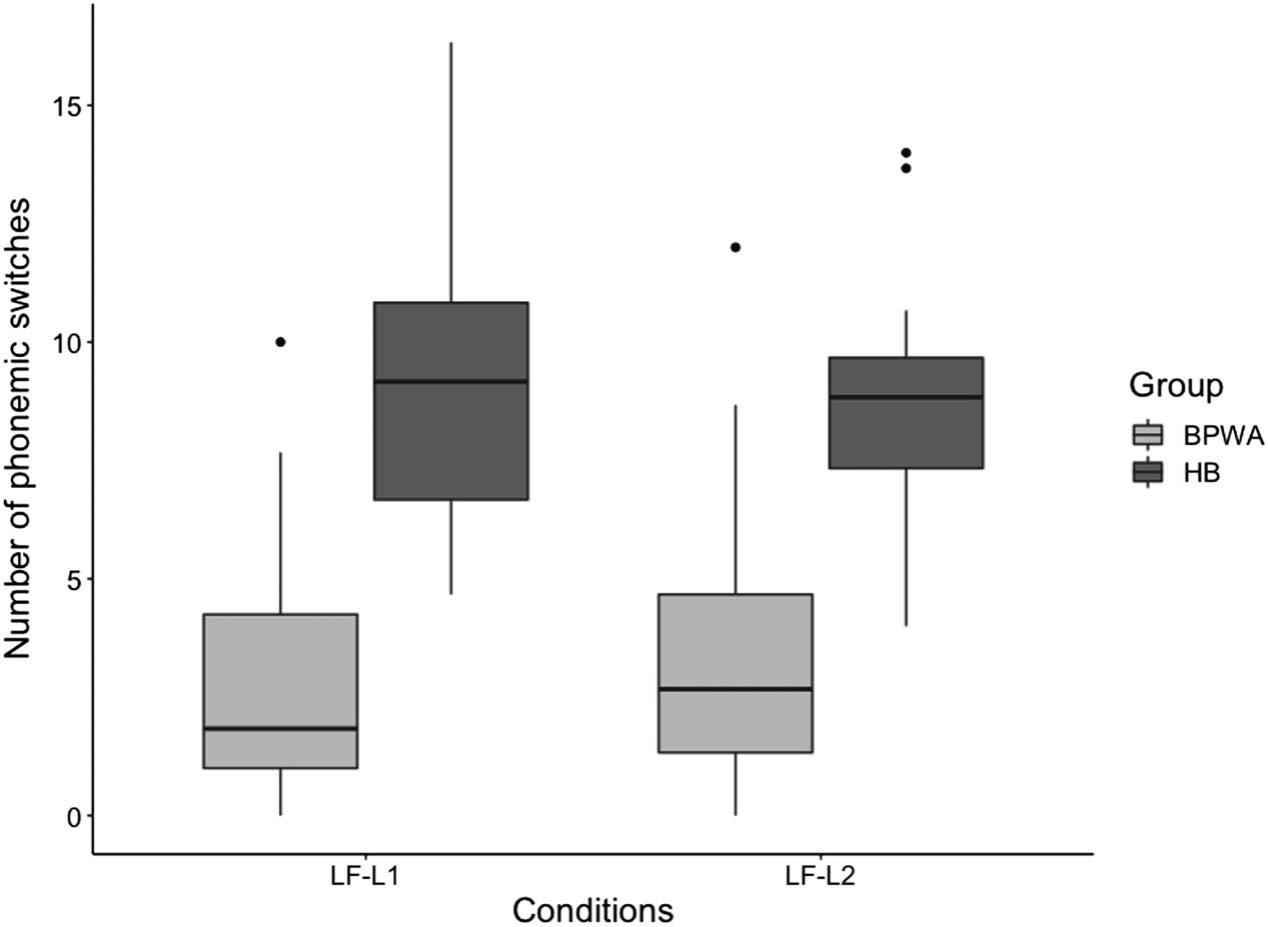
The number of phonemic switches produced by the bilingual patients with aphasia (BPWA) and healthy bilinguals (HB) on the letter fluency task. Boxplots are shown separately for each group across the two conditions (LF-L1 and LF-L2). BPWA showed significantly fewer phonemic switches relative to the HB in both conditions.

Results from the repeated-measures ANOVA revealed a significant main effect of Group [*F*(1, 54) = 76.290, *p* < 0.001, *η*_*p*_^2^ = 0.568], although the main effect of Condition [*F*(1, 54) = 0.349, *p* = 0.557, *η*_*p*_^2^ = 0.006] and the Group × Condition interaction were not significant [*F*(1, 54) = 1.140, *p* = 0.290, *η*_*p*_^2^ = 0.021], indicating that BPWA produced fewer phonemic switches than healthy bilinguals and groups demonstrated similar performance across conditions (Figure 7). Independent samples *t* tests revealed that BPWA produced significantly fewer switches in both conditions (*p-*adj < 0.001 in both cases).

### Factors That Predict Performance on FS and SS

For BPWA, the PCA conducted for L1 revealed three components with eigenvalues greater than 1 that explained 72% of the variance. Using a factor loading threshold of 0.6, it was determined that Component 1 represents L1 Background, Component 2 represents L1 Use and Exposure, and Component 3 represents L1 Environment. A varimax normalized factor rotation was used to examine the factor loadings for the first three components. The PCA conducted for L2 revealed two components with eigenvalues greater than 1 that explained 63% of the variance. The factor loadings for L2 indicate that Component 1 represents L2 Background and Environment, and Component 2 represents L2 Use and Exposure. Again, a varimax normalized factor rotation was used to examine the factor loadings for the first two components. (Table 5).

**Table 5.** Factor loadings for L1 and L2 Language Use Questionnaire totals for BPWA.

	**L1**			**L2**	
	**RC 1 (Background)**	**RC 2 (Use/Exposure)**	**RC 3 (Environment)**	**RC 1 (Background/Environment)**	**RC 2 (Use/Exposure)**
L2 AoA	–	–	–	**−0.84**	−0.17
Pre-ABI LAR	**0.85**	0.23	−0.01	0.46	**0.63**
Post-ABI LAR	0.46	−0.08	0.58	0.01	0.58
Pre-ABI use	−0.02	**0.90**	−0.05	0.15	**0.75**
Post-ABI use	0.29	**0.83**	0.14	0.15	**0.77**
Family proficiency	−0.08	0.18	**0.89**	**0.86**	0.07
Education	**0.74**	0.41	0.08	0.50	0.58
Exposure	0.50	**0.68**	0.21	0.52	**0.69**
Confidence	**0.72**	0.03	0.08	**0.85**	0.19
Variance	29%	28%	15%	33%	31%

*Note*. Factor loadings greater than 0.6 are in bold. L2 AoA = L2 age of acquisition; ABI = acquired brain injury; LAR = language ability rating; RC = rotated component.

For healthy bilinguals, the PCA conducted for L1 revealed one component with an eigenvalue greater than 1 that explained 50% of the variance. Again, using a threshold of 0.6, the factor loadings indicate that Component 1 represents L1 Background and Exposure. A varimax normalized factor rotation was used to examine which factors loaded ono this component. The PCA conducted for L2 revealed two components with eigenvalues greater than 1 that explained 72% of the variance. The factor loadings for L2 indicate that Component 1 represents L2 Background and Environment, and Component 2 represents L2 Use and Exposure. Again, a varimax normalized factor rotation was used to examine which factors loaded onto these components (Table 6).

**Table 6.** Factor loadings for L1 and L2 Language Use Questionnaire totals for healthy bilinguals.

	**L1**	**L2**	
	**RC 1 (Background/Exposure)**	**RC 1 (Background/Environment)**	**RC 2 (Use/Exposure)**
L2 AoA	–	**−0.81**	−0.17
LAR	**0.84**	0.21	**0.83**
Use	0.50	−0.05	**0.77**
Family proficiency	0.39	**0.80**	−0.04
Education	**0.94**	**0.64**	0.58
Exposure	**0.81**	0.58	**0.72**
Confidence	**0.81**	**0.69**	0.58
Variance	50%	37%	36%

*Note*. Factor loadings greater than 0.6 are in bold. AoA = age of acquisition; LAR = language ability ratings; RC = rotated component.

As Table 7 shows, backward stepwise regressions were conducted for each of the measures for BPWA. Results of the first multiple linear regression including PAPT, L1 BAT, L1 Use and Exposure, and L2 Background and Environment explained 64% of the variance of SS cluster size in BPWA [*F*(4, 8) = 6.251, *p* = 0.014], with L1 BAT as the only significant predictor in the model. Results of the second multiple linear regression including L2 BAT, RCPM, L1 Use and Exposure, L2 Background and Environment, and L2 Use and Exposure explained 71% of the variance of SS switches [*F*(5, 7) = 6.738, *p* = 0.013], with L2 BAT, L1 Use and Exposure, L2 Background and Environment, and L2 Use and Exposure significantly predicting SS switches; however, L2 Background and Environment did not survive corrections for multiple comparisons. Results of the third multiple linear regression including PAPT, L1 BAT, L2 BAT, RCPM and L1 Environment explained 50% of the variance of FS cluster size [*F*(5, 7) = 3.355, *p* = 0.073]; however, no individual predictors were significant (*p* values > 0.05 in all cases). Results of the fourth multiple linear regression including PAPT, L1 BAT, L2 BAT, L1 Background, L2 Background and Environment, and L2 Use and Exposure explained 33% of the variance of FS switches [*F*(6, 6) = 1.964, *p* = 0.216], with PAPT, L1 Background, L2 Background and Environment, and L2 Use and Exposure significantly predicting FS switches.

**Table 7.** Multiple linear regression results for BPWA.

**Dependent variable**	**Independent variable**	***B***	***SE***	***t***	***P*r (> |*t*|)**	***p*-adj**
SS cluster size	(Intercept)	1.278	1.564	0.817	0.438	0.438
	PAPT	−2.684	1.969	−1.363	0.210	0.263
	**L1 BAT**	**3.478**	**0.941**	**3.697**	0.006**	0.030*
	L1 Component 2 (Use/Exposure)	−0.544	0.255	−2.130	0.066	0.137
	L2 Component 1 (Background/Environment)	0.595	0.300	1.987	0.082	0.137
SS switches	(Intercept)	4.724	2.240	2.109	0.073	0.087
	**L2 BAT**	**6.042**	**2.098**	**2.880**	0.024*	0.047*
	RCPM	−5.465	3.173	−1.722	0.129	0.129
	**L1 Component 2 (Use/Exposure)**	**−3.673**	**0.773**	**−4.753**	0.002**	0.013*
	**L2 Component 1 (Background/Environment)**	**1.280**	**0.497**	**2.577**	0.037*	0.055
	**L2 Component 2 (Use/Exposure)**	**−3.459**	**0.905**	**−3.823**	0.007**	0.020*
FS cluster size	(Intercept)	−2.063	2.782	−0.742	0.482	0.482
	PAPT	8.248	5.069	1.627	0.148	0.296
	L1 BAT	4.680	2.846	1.645	0.144	0.296
	L2 BAT	3.715	2.887	1.287	0.239	0.349
	RCPM	−11.672	5.352	−2.181	0.066	0.296
	L1 Component 3 (Environment)	0.377	0.330	1.143	0.291	0.349
FS switches	**(Intercept)**	**16.089**	**4.672**	**3.443**	0.012*	0.050*
	**PAPT**	**−17.338**	**6.018**	**−2.881**	0.028*	0.050*
	L1 BAT	8.273	4.191	1.974	0.096	0.112
	L2 BAT	−7.574	4.146	−1.827	0.118	0.117
	**L1 Component 1 (Background)**	**2.611**	**0.968**	**2.697**	0.036*	0.050*
	**L2 Component 1 (Background/Environment)**	**2.690**	**0.858**	**3.134**	0.020*	0.050*
	**L2 Component 2 (Use/Exposure)**	**2.451**	**0.863**	**2.839**	0.030*	0.050*

*Note*. All significant results are marked in bold. SS = Self-Switch; FS = Forced-Switch; L1 BAT = Bilingual Aphasia Test, L1; L2 BAT = Bilingual Aphasia Test, L2; PAPT = Pyramids and Palm Trees; RCPM = Raven’s Coloured Progressive Matrices. *p*-adj = adjusted *p* value. *p < 0.05; **p < 0.01. Adjusted *p* values were calculated via the Benjamini Hochberg procedure using the Multcomp package in R (https://cran.r-project.org/web/packages/multcomp/index.html).

For the healthy bilinguals, Table 8 shows results of the first multiple linear regression including L2 Background and Environment that explained 7% of the variance of SS cluster size [*F*(1, 20) = 2.558, *p* = 0.125], and no individual predictors were significant. Results of the second multiple linear regression including PAPT, L1 Background and Exposure, and L2 Background and Environment explained 50% of the variance of SS switches [*F*(3, 18) = 7.983, *p* = 0.001], with PAPT significantly predicting SS switches. Results of the third multiple linear regression including PAPT and L1 BAT explained 7% of the variance of FS cluster size [*F*(2, 19) = 1.782, *p* = 0.195], and no individual predictors were significant. Results of the fourth multiple linear regression including L1 BAT scores explained 17% of the variance of FS switches [*F*(1, 20) = 7.299, *p* = 0.033], with L1 BAT significantly predicting FS switches; however, this did not survive corrections for multiple comparisons.

**Table 8.** Multiple linear regression results for healthy bilinguals.

**Dependent variable**	**Independent variable**	***B***	***SE***	***t***	***P*r (> |*t*|)**	***p*-adj**
SS cluster size	**(Intercept)**	**1.995**	**0.139**	**14.334**	<0.001***	<0.001***
	L2 Component 1 (Background/Environment)	0.250	0.156	1.599	0.125	0.125
SS switches	**(Intercept)**	**−18.343**	**6.170**	**−2.973**	0.008**	0.0163*
	**PAPT**	**27.133**	**6.497**	**4.176**	<0.001***	0.002**
	L1 Component 1 (Background/Exposure)	0.838	0.438	1.911	0.072	0.087
	L2 Component 1 (Background/Environment)	−0.790	0.436	−1.811	0.087	0.087
FS cluster size	(Intercept)	−2.929	3.238	−0.905	0.377	0.377
	PAPT	9.486	5.111	1.856	0.079	0.163
	L1 BAT	−4.478	2.660	−1.683	0.109	0.163
FS switches	(Intercept)	−1.804	3.730	−0.484	0.634	0.634
	**L1 BAT**	**9.176**	**3.995**	**2.297**	0.033*	0.065

*Note*. All significant results are marked in bold. SS = Self-Switch; FS = Forced-Switch; L1 BAT = Bilingual Aphasia Test, L1; L2 BAT = Bilingual Aphasia Test, L2; PAPT = Pyramids and Palm Trees (Howard & Patterson, 1992). *p < 0.05; **p < 0.01; ***p < 0.001. Adjusted *p* values were calculated via the Benjamini Hochberg procedure using the Multcomp package in R (https://cran.r-project.org/web/packages/multcomp/index.html).

## DISCUSSION

The purpose of this study was to examine how varying degrees of cognitive control demands on lexical retrieval impacted performance in two verbal fluency tasks in healthy bilinguals and BPWA using four language contexts. The conditions implemented in the semantic category generation task included two No-Switch conditions (NS-L1 and NS-L2), where participants responded only in one language, one Self-Switch condition (SS), where participants switched between languages as desired, and one Forced-Switch condition (FS), where participants were required to switch languages after each response. Additionally, participants completed a traditional letter fluency task in each language separately (LF-L1 and LF-L2). Overall, we found that healthy bilinguals, in general, outperformed BPWA across all measures. This is consistent with previous research (Carpenter et al., 2020; Kiran et al., 2014; Patra et al., 2020a) and reflects varying degrees of language impairment in the BPWA group. First, we examined FDSs in L1 and L2 as a measure of cognitive control abilities and found that both groups demonstrated smaller FDSs (indicative of better control) in L1 compared to L2. Our second aim examined semantic clustering and switching performance across the four conditions of the semantic category generation task. No group differences were found in mean semantic cluster size across the four conditions. Additionally, while healthy bilinguals did not differ in the number of switches produced across conditions, BPWA produced more switches in SS compared to FS and NS-L1 compared to NS-L2 and FS. Our third aim examined phonemic clustering and switching performance in the two conditions of the letter fluency task. The results of this analysis showed group differences in mean phonemic cluster sizes produced in the two conditions, indicating that healthy bilinguals performed better in LF-L1 compared to LF-L2 while BPWA showed similar performance across conditions. Additionally, no differences were observed in the number of switches between the two conditions for either group. Finally, our fourth aim examined which language experience measures (LUQ metrics) and standardized assessment scores predicted switching and clustering performance in the two dual-language conditions (SS and FS) for both groups separately. For both groups, switching performance was more dependent on language factors than clustering, with BPWA switching performance relying on both language experience and standardized assessment scores, while healthy bilingual performance relied solely on standardized assessment scores. The main findings of this study are reported in Table 9.

**Table 9.** Summary of main findings.

**Measure**	**Between group effects**	**Across condition effects**
Fluency difference scores	HB > BPWA	L1 > L2
Mean semantic cluster size	HB > BPWA (except FS)	No differences
Number of semantic switches	HB > BPWA in all conditions	SS > FS; NS-L1 > NS-L2 and FS for BPWA only
Mean phonemic cluster size	HB > BPWA in LF-L1	LF-L1 > LF-L2 for HB only
Number of phonemic switches	HB > BPWA in both conditions	LF-L1 = LF-L2 for HB and BPWA
SS clusters	Predicted by standardized assessment scores for BPWA.	
SS switches	Predicted by standardized assessment scores for HB and BPWA. Predicted by language experience measures for BPWA.	
FS clusters	Not predicted by any measures for either group.	
FS switches	Predicted by standardized assessment scores for HB and BPWA. Predicted by language experience measures for BPWA.	

*Note*. BPWA = bilingual patients with aphasia; HB = healthy bilinguals; SS = Self-Switch; NS-L1 = No Switch (L1); NS-L2 = No Switch (L2); FS = Forced-Switch.

First, in regard to cognitive control abilities across L1 and L2, we found that healthy bilinguals demonstrated better control than BPWA overall, as evidenced by smaller FDSs. Additionally, both groups demonstrated superior performance in L1 compared to L2. This was anticipated, as better control demands in L1 are reflective of less inhibitory processes required to suppress the less-dominant L2. For BPWA, these results are consistent with reduced switching performance observed in NS-L2 compared to NS-L1, as in this condition BPWA are less able to manage increased control demands arising from the level of language control in order to inhibit their more dominant L1, leading to reduced ability to systematically search within the lexicon for new lexical candidates.

Second, in line with previous research (Kiran et al., 2014), BPWA showed smaller mean semantic cluster sizes and number of switches compared to the healthy bilinguals. Of note, both groups did not differ in terms of mean semantic cluster size produced across the four conditions of the semantic category generation task. This comparable clustering performance across the four conditions was expected, as clustering reflects the automatic process of spreading activation and therefore is less likely to be impacted by increased language control demands. Second, when examining switching performance, differences were observed in number of switches produced across the four conditions. In particular, while healthy bilinguals demonstrated comparable switching performance across the four conditions, BPWA produced more switches in the SS condition compared to FS condition, as well as in the NS-L1 compared to the NS-L2 and FS conditions. These results highlight the interaction between language control and semantic executive control underlying lexical retrieval across the different conditions. Specifically, in the FS condition (Figure 1C), participants are tasked with switching between languages for each new item produced, which requires speakers to inhibit competition across two levels of control, language control and semantic executive control. Results indicate that the increased top-down inhibitory control demands arising from the level of language control in order to inhibit the previously activated language, impedes BPWA’s abilities to implement semantic executive control processes needed for successful semantic switching performance. Furthermore, BPWA showed reduced switching performance in the NS-L2 condition also, highlighting that BPWA may have more difficulty resolving conflict at the language control level in their weaker language (Figure 1A). Specifically, compared to the NS-L1 condition, in the NS-L2 condition, speakers are required to use larger amounts of inhibitory processes at the language control level in order to suppress the dominant L1 in favor of the weaker L2. For BPWA, damage to the language system reduces the amount of cognitive resources they are able to dedicate to each level of control; therefore in the NS-L2 condition, the increased resources being allocated to the language control level reduces the ability of BPWA to implement the controlled search mechanisms required to initiate a new cluster. Overall, these results are consistent with findings reported by Troyer and colleagues (1997), where a divided attention condition led to a reduced number of switches but not clustering, reinforcing that switching and clustering are dissociable components of verbal fluency tasks and increased cognitive demands may impact controlled processes (switching) more than automatic processes (clustering). Additionally, for individuals with focal brain damage, reduced cognitive resources leads to worse performance not only in the condition with the highest cognitive control demands (i.e., FS), but also in the condition with moderate cognitive control demands (i.e., NS-L2).

Third, in the letter fluency task, we found again that healthy bilinguals overall produced larger mean phonemic cluster sizes and switched more times compared to BPWA. Interestingly, the interaction effect observed for these mean phonemic cluster sizes indicated that only healthy bilinguals produced larger phonemic cluster sizes in L1 compared to L2, although neither group differed in number of switches produced in either language. Performance of the healthy bilinguals directly contrasts the results of the semantic category generation task, perhaps reflecting the distinctions in search mechanisms on letter fluency tasks compared to semantic category generation tasks. As noted in the Introduction, during phonological retrieval in letter fluency tasks, suppression of semantic competition in favor of phonologically related words requires more controlled processes rather than reliance on automatic activation of related concepts (Figure 2). These increased processing demands would be further impacted by proficiency as the L2 phonological system is generally less well developed than the L1 phonological system (Dijkstra & van Heuven, 1998, 2002; Dijkstra et al., 2019; Kroll & Stewart, 1994), leading to less readily available lexical representations. Since the healthy bilinguals demonstrated larger mean phonemic cluster sizes in the LF-L1 condition compared to the LF-L2, but BPWA did not, it appears that the BPWA were more sensitive to the increased demands imposed by this task. While healthy bilinguals were able to make greater use of semantic executive control processes to systematically search within the lexicon for phonemically related concepts in L1, where there is less competition arising from the level of language control, BPWA demonstrated reduced clustering performance in both L1 and L2.

Finally, the regression analyses revealed differences in factors that contributed to switching and clustering performance across conditions and groups. First, for the BPWA, L1 BAT scores significantly contributed to SS clustering performance indicating that when language is not a constraint, clustering performance solely depends on language abilities (as measured by standardized assessments), rather than language experience. Second, L2 BAT scores, L1 Use and Exposure, and L2 Use and Exposure significantly contributed to SS number of switches produced. These results indicate that the higher an individual’s L2 BAT scores the more they switched between semantic subcategories, suggesting that greater semantic ability aids in systematically searching the mental lexicon for new lexical candidates once a semantic subcategory has been exhausted. Additionally, the negative slopes for Use and Exposure in L1 and L2 suggest that as language experience increases, the number of semantic switches produced by BPWA declines. This indeed may indicate that individuals with greater language experience are able to further extend semantic subcategories, therefore reducing the need to switch.

Further, FS clustering performance was not predicted by any standardized assessment scores or language experience measures. This finding was surprising, as it was expected that better performance on standardized assessments would lend itself to clustering performance when cognitive demands increased. However, it may indeed be the case that the more errors and overall fewer responses elicited by this condition may contribute to a floor effect in the data. Finally, FS switching performance was significantly predicted by PAPT scores, L1 Background, L2 Background and Environment, and L2 Use and Exposure. These results indicate that greater nonverbal semantic knowledge (PAPT scores) and language experience in both languages benefits switching performance when the language demands of the task increase. More specifically, semantic switching in the FS condition places the highest control demands on both language control and semantic executive control (see Figure 1C and Figure 2). BPWA, who present with reduced cognitive resources to allocate to different levels of control, are less able to manage competition between language control and semantic executive control. Therefore, within this group, it may be possible that their L1 and L2 language experience aids in compensating for these deficits by dedicating fewer cognitive resources to language control, allowing them to better implement semantic executive control processes required when attempting to switch between clusters.

For the healthy bilinguals, SS and FS clustering was not predicted by standardized assessment scores or language experience measures. This finding may relate to the fact that when there are no language constraints, automatic spreading activation and not language experience may drive the outcome. Second, PAPT scores significantly predicted switching performance on the SS condition underscoring the notion that nonverbal semantic knowledge (as measured by PAPT) lends itself to more readily available items when switching subcategories. Finally, FS switching was not predicted by any measures after correcting for multiple comparisons.

Of note, some interesting patterns arose when looking at the regression results across the two groups. First, switching performance, regardless of condition was dependent on standardized assessment scores for both healthy bilinguals and BPWA, whereas clustering performance was not predicted by standardized assessment scores or language experience measures. These results suggest that the controlled search processes required of switching are highly dependent on the strength of semantic and lexical representations and access to them. Notably, BPWA switching performance was dependent on language experience measures in the FS condition. This indicates that for BPWA, switching performance when language demands of the task increase relies on both standardized assessment scores and language experience in L1 and L2. This may underscore the relationship between impairment and language proficiency in BPWA and the importance of language control for successful production, as reduced language control abilities lead to greater competition with semantic executive control processes, therefore disrupting lexical production in BPWA. BPWA with greater language experience in L1 and L2 may be better at resolving conflict at the level of language task schemas in order to implement controlled semantic executive control processes for better semantic switching performance. This is consistent with the findings of Carpenter et al. (2020), which highlights that increased language control demands may, at least in part, lead to reduced communicative success in this population. This highlights the importance of understanding language control in BPWA and how it may contribute to more functional communication outcomes post-ABI.

One limitation of this study is that for BPWA, RCPM performance did not make a significant contribution to any of the predictive models, suggesting that RCPM, a measure of matrix reasoning (Fong et al., 2020), may not sufficiently tap the executive functions intrinsically related to the mechanisms used in switching and clustering performance. Future studies should carefully select measures of executive functions as to better underscore the mechanisms used across the different conditions. Of note, this study consisted of primarily Spanish dominant Spanish-English bilinguals. However, follow-up analyses to examine whether there was an effect of participants’ reported L1 on their verbal fluency performance found no differences in switching and clustering scores when separately grouping individuals by their reported L1 (Spanish or English). This was consistent when examining just the BPWA group. While there were fewer L1 English individuals in this study, the lack of differences suggests that both groups were performing comparably with the L1 Spanish individuals across the four conditions, suggesting these results may be generalizable to English dominant Spanish–English bilinguals. Additionally, healthy bilinguals had higher education overall compared to BPWA, which may influence their performance on verbal fluency tasks; future studies may consider using education as a covariate when investigating verbal fluency performance in similar populations. Finally, lower verbal fluency scores for the BPWA in general may have contributed to a potential floor effect in the data. However, reanalysis of the patient data including only individuals with higher verbal fluency scores did not change the overall results.

### Conclusions

The present findings suggest that switching and clustering are dissociable components of verbal fluency performance, with switching being more sensitive to the impact of cognitive control demands. Specifically, as language demands of the task increased, BPWA’s abilities to implement semantic executive control was hindered. Additionally, switching and clustering performance may differ on semantic category generation tasks as compared to letter fluency tasks due the underlying mechanisms required of each task. Furthermore, language abilities may play a greater role in the ability to implement controlled processes (switching) rather than automatic ones (clustering) for both healthy bilinguals and BPWA. Importantly, for BPWA standardized assessment scores as well as language experience in L1 and L2 greatly influence semantic switching abilities when language demands of the task increase, highlighting the importance of language control for successful lexical production post-ABI.

## ACKNOWLEDGMENTS

This work was supported by the National Institute on Deafness and Other Communication Disorders of the National Institutes of Health (grant U01DC014922) awarded to Swathi Kiran. The content is solely the responsibility of the authors and does not necessarily represent the official views of the National Institutes of Health.

## FUNDING INFORMATION

Swathi Kiran, National Institute on Deafness and Other Communication Disorders (https://dx.doi.org/10.13039/100000055), Award ID: U01DC014922.

## AUTHOR CONTRIBUTIONS

**Erin Carpenter**: Data curation: Equal; Formal analysis: Lead; Methodology: Equal; Writing – original draft: Lead; Writing – review & editing: Equal. **Claudia Peñaloza**: Conceptualization: Equal; Data curation: Equal; Investigation: Equal; Methodology: Equal; Project administration: Equal; Writing – review & editing: Supporting. **Leela Rao**: Conceptualization: Equal; Data curation: Equal; Methodology: Equal. **Swathi Kiran**: Conceptualization: Lead; Funding acquisition: Lead; Investigation: Lead; Methodology: Lead; Project administration: Lead; Writing – review & editing: Lead.

## COMPETING INTERESTS

Swathi Kiran serves as a consultant for Constant Therapy Health with no scientific overlap with the present study. All other authors have no financial or nonfinancial conflicts of interest.

## TECHNICAL TERMS

Language control: The management of competition from language task schemas via inhibitory control mechanisms for appropriate production of the intended language.
Semantic executive control: The use of control mechanisms that direct activation and inhibition of lexical and phonological representations at the lexical level.
Aphasia: An acquired language disorder affecting language comprehension and production following acquired brain injury.
